# Abnormally elevated expression of ACTA2 of circular smooth muscle leads to hyperactive contraction in aganglionic segments of HSCR

**DOI:** 10.1007/s00383-023-05479-x

**Published:** 2023-06-06

**Authors:** Ke Chen, Jingyi You, Shimin Yang, Xinyao Meng, Xuyong Chen, Luyao Wu, Xiaosi Yu, Jun Xiao, Jiexiong Feng

**Affiliations:** 1 Department of Pediatric Surgery, Tongji Hospital, Tongji Medical College of Huazhong University of Science and Technology, 1095 Jiefang Ave, Wuhan, 430043 China; 2 Hubei Clinical Center of Hirschsprung Disease and Allied Disorders, Wuhan, China

**Keywords:** Hirschsprung disease, Circular and longitudinal muscles, ENCCs, ACTA2, Embryonic development

## Abstract

**Background:**

Actin Alpha 2 (ACTA2) is expressed in intestinal smooth muscle cells (iSMCs) and is associated with contractility. Hirschsprung disease (HSCR), one of the most common digested tract malformations, shows peristaltic dysfunction and spasm smooth muscles. The arrangement of the circular and longitudinal smooth muscle (SM) of the aganglionic segments is disorganized. Does ACTA2, as a marker of iSMCs, exhibit abnormal expression in aganglionic segments? Does the ACTA2 expression level affect the contraction function of iSMCs? What are the spatiotemporal expression trends of ACTA2 during different developmental stages of the colon?

**Methods:**

Immunohistochemical staining was used to detect the expression of ACTA2 in iSMCs of children with HSCR and *Ednrb*^*−/−*^ mice, and the small interfering RNAs (siRNAs) knockdown technique was employed to investigate how *Acta2* affected the systolic function of iSMCs. Additionally, *Ednrb*^*−/−*^ mice were used to explore the changes in the expression level of iSMCs ACTA2 at different developmental stages.

**Results:**

The expression of ACTA2 is higher in circular SM in the aganglionic segments of HSCR patients and *Ednrb*^*−/−*^ mice than in normal control children and mice. Down regulation of *Acta2* weakens the contraction ability of intestinal smooth muscle cells. Abnormally elevated expression of ACTA2 of circular smooth muscle occurs since embryonic day 15.5 (E15.5d) in aganglionic segments of *Ednrb*^*−/−*^ mice.

**Conclusions:**

Abnormally elevated expression of ACTA2 in the circular SM leads to hyperactive contraction, which may cause the spasm of aganglionic segments in HSCR.

**Supplementary Information:**

The online version contains supplementary material available at 10.1007/s00383-023-05479-x.

## Introduction

Hirschsprung disease (HSCR) is one of the most common malformations of the digestive tract, with an incidence of 1 in 5,000 live births. Patients with HSCR frequently manifest severe constipation and abdominal distension, which require surgical removal of the pathological segments of the distal bowel [1–3]. In addition, HSCR patients could suffer severe complications such as HAEC [4, 5]. The primary pathological feature of HSCR is the absence of ganglion cells in the distal bowel submucosal and myenteric plexuses. Furthermore, the arrangement of the circular and longitudinal smooth muscle (SM) of the aganglionic segments is disorganized [6]. Our previous study discovered that aganglionic segments of HSCR model mice (*Ednrb*^*−/−*^) displayed peristaltic dysfunction [4], and the smooth muscle of the aganglionic colons would spasm [7, 8].

The protein encoded by Actin Alpha 2 (ACTA2), also known as alpha smooth muscle actin (α-SMA), belonging to the actin protein family, is a highly conserved protein that plays a role in cell motility, structure, and integrity. ACTA2 is widely expressed in visceral and vascular SM and involved in contraction [9]. Studies have shown that ACTA2 expressed in intestinal smooth muscle cells (iSMCs) [10–14]. David Fawkner Corbett and Tyler R. Huycke indicated that ACTA2 is a maker to observe the development of fetal iSMCs, respectively [12, 15–18]. Hongyu Qiu et al. found that, in the vascular smooth muscles cells, the expression level of ACTA2 increased, resulting in more contractile [19]. In Mohamed M Ibrahim’s study, they found that fibroblasts from Acta2^−/−^ mice had diminished contractility, resulting in slower wound contraction closure [20]. In iSMCs, we speculated that the expression of ACTA2 is also associated with contractility.

After birth, the smooth muscle morphology of the aganglionic segment was disordered, the contractile function was abnormal. Dose ACTA2, an iSMCs marker, exhibits abnormal expression? Does the ACTA2 expression level affect the contractility of iSMCs? ENCCs proliferation and migration are regulated by genetic and environmental factors throughout the embryonic phase of HSCR patients, leaving the distal segment devoid of enteric neurons [21–23]. ENCCs have an impact on the differentiation of mesenchymal stem cells into iSMCs [24]. It's possible that in the absence of ENCCs, mesenchymal cells cannot differentiate into normal iSMCs. Is there an abnormal development of iSMCs in the aganglionic segment of HSCR during the embryonic period? In order to investigate the aforementioned issues, expression of ACTA2 was detected by immunohistochemical staining in iSMCs of children with HSCR and *Ednrb*^*−/−*^ mice, and the small interfering RNAs (siRNAs) knockdown technique was employed to investigate how ACTA2 affected the systolic function of iSMCs. Additionally, *Ednrb*^*−/−*^ mice were used to explore the changes in the expression level of iSMCs ACTA2 at different developmental stages.

## Methods

### Collection of samples

Thirty pathological samples were collected from HSCR patients undergoing surgery at Tongji Hospital, Huazhong University of Science and Technology, in the years 2020–2022, as well as five normal tissues from patients injured in car accidents. All patients (or their legal guardians) must sign an informed consent form before using clinical specimens. The ethics committee of Tongji Hospital approved the use of the organization for this study (Ethics approval number: 2020-S226). The Jackson Laboratory made breeding populations of Ednrb^tm1Ywa/J^ hybrid mice (Ednrb^tm1Ywa/J^ in a C57BL/6J-129 Sv hybridization background) available (JAX-003295). The WT and Ednrb hybrid mice aged 6–8 weeks were fed in cages with a female ratio of 2:1, respectively. The distal colons of WT and *Ednrb*^*−/−*^ mice were collected at E12.5d, E13.5d, E15.5d, E17.5d, E19.5d, postnatal days 1 (P1d), P7d, and P21d, respectively, at the different developmental stages. The Animal Care and Use Committee at Tongji Hospital approved the animal research program (Ethics Approval Number: TJH-202111024).

### H&E staining

After removing the paraffin, tissue sections were hydrated with gradient ethanol, stained with hematoxylin solution for nuclear staining and other possible acidic cellular components, washed with running water, differentiated with acid alcohol to remove nonspecific hematoxylin staining from slides and tissues, followed by water rinsing, and slides were immersed in ammonia to change the hematoxylin-stained nuclei from a red to a blue-purple appearance. After dehydration with gradient ethanol, the tablets were counterstained with eosin, dehydrated with absolute ethanol, and sealed with xylene replacement after transparency.

### Immunohistochemical analysis

After being deparaffinized in xylene and rehydrated using a graduated ethanol series, paraffin-embedded sections of colon tissue were microwaved for 16 min with a tris–EDTA antigen retrieval solution. Incubated 3% H_2_O_2_ at room temperature for 5–10 min to eliminate the activity of endogenous peroxidase. To block non-specific binding sites, slides were incubated with 5% bovine serum albumin (BSA) and 0.1% Triton X-100 in phosphate-buffered saline (PBS) for 1 h at 37 °C. Next, ACTA2 antibody (1:1000, Abcam, ab7817) was added, which was then incubated overnight at 4 °C before being followed by horseradish peroxidase anti-mouse IgG antibody for 1 h. The DAB substrate kit (Servicebio) was then incubated with the slides to develop the colors. Under a powerful microscope, each diseased segment was examined in five randomly chosen fields. The appearance of brown or yellowish-brown granular particles in the cytoplasm was deemed a sign of positive expression of ACTA2. Under the blind principle, three experienced pathologists independently evaluated the experimental results. The staining intensity was graded on a four-point scale: 0 = negative, 1 = weak, 2 = medium, and 3 = strong. The scoring result was expressed by the staining intensity multiplied by the percentage of positive cells. The highest intensity was used for scoring if the staining intensity was uneven in the area; the estimated percentage of stained SMCs was divided into 1 (0–50%), 2 (51–70%), 3 (71–90%), and 4 (> 90%). To determine the intensity of specific staining, Image Pro Plus 6.0 software (Media Cybernetics, Silver Spring) was used. All images were captured using the same microscope and camera unit, and the average IOD (IOD/Area) of each positive staining area was calculated [25–27].

### Isolation and culture of primary iSMCs

C57BL/6J fetuses were retrieved via cesarean section on E15–18.5d and kept in precooled PBS. The intestines were removed from the abdomen and placed in PBS. Smooth muscle cells were isolated from intestines using previously reported methods of enzymatic digestion, filtration, and centrifugation [28–30]. After that, the tube was placed in a 37 °C cell culture incubator, digested for 30 min, and blown once every 10 min. Add an equal volume of DMEM culture medium (Gibco) to stop digestion. After being filtered and centrifuged, the iSMCs were resuspended in a 60-mm plastic dish with 3 ml of DMEM containing 10% fetal bovine serum (FBS, Gibco), and the dish was placed at 37 °C in a humidified environment with 5% CO2. The cultural media were replaced every 2-3 days.

### Immunofluorescence

For immunohistochemical staining of iSMCs, the cells on the slides were fixed with 4% paraformaldehyde for 30 min at room temperature and then washed three times with PBS. After 30 min of blocking with 5% BSA and 0.1% Triton X-100, the primary antibody (1:1000, abcam, ab7817) was added, incubated overnight at 4 °C and rinsed three times with PBS. After 1 h of incubation with the secondary antibody, the sample was rinsed three times with PBS, DAPI and a sealing agent were added dropwise to seal the sample, and images were taken using a fluorescence microscope (DM6 B, Leica).

### siRNA transfection

To obtain a fusion proportion of 30–50%, inoculated 1 × 10^6^ cells per well in 6-well plates were inoculated 24 h before transfection. 10 μl transfection reagent was diluted with 250 μl Opti-MEM, and 5 μl siRNA was diluted with 250 μl Opti-MEM. The diluted transfection reagent and siRNA were combined, and the combination was placed in a 6-well plate. After 4 h of incubation at 37 °C and 5% CO2, 1500 μl complete medium was added to each well of the cell plate and cultivated for 48 h.

### Quantitative RT-PCR

The primary-cultured iSMCs inoculated in 6-well plates were treated with trizol (Vazyme) to isolate total RNA. After determining the RNA concentration, reverse transcription can be performed using HiScript III RT SuperMix for qPCR (Vazyme). To measure particular cDNA, ChamQ Universal SYBR qPCR Master Mix (Vazyme) and the CFX Connect Real-Time PCR Detection System (Bio-Rad) were used. The data are presented as a relative expression index in comparison to the control sample.

### Western blot analysis

After total protein extraction from iSMCs, the proteins were measured using the BCA protein assay (Servicebio). A Bis–Tris gel (ACE Biotechnology) was filled with fresh extracts. Proteins were then transferred to a polyvinylidene fluoride membrane and blocked with 5% BSA. An anti-ACTA2 antibody (1:3000, Abcam, ab7817) was used to test ACTA2 expression. Secondary antibodies coupled to horseradish peroxidase were used to detect the signals.

### Collagen gel contraction assay

To create a working solution for a neutral pH gel, prepared 1 M NaOH, ddH2O, 10 × PBS, and rat tail collagen I (Corning). Combined these ingredients in a precooled sterile centrifuge tube. Blended the cell suspension with the working gel solution in a 1:4 ratio. Then added 500 μl of the aforementioned mixture to each well of a 24 well plate and placed in a 37 °C cell incubator for 1 h, or until the mixture hardened. Removed the gel off the edge of the 24-well plate with a scraper, placed it in the 6-well plate well that had been filled with complete culture medium, cultured it for 2 days at 37 °C, took images at 24 and 48 h, and calculated the gel area.

### Statistics

Standard deviations as well as means were provided for all outcomes. The differences between the two groups were examined using two-tailed Student’s *t* tests, with *p < *0.05 being considered statistically significant. Each test was repeated at least thrice.

## Results

### The expression of ACTA2 is higher in circular SM in the aganglionic segments of HSCR patients and Ednrb^−/−^ mice than in normal control children and mice

**Fig. 1:**
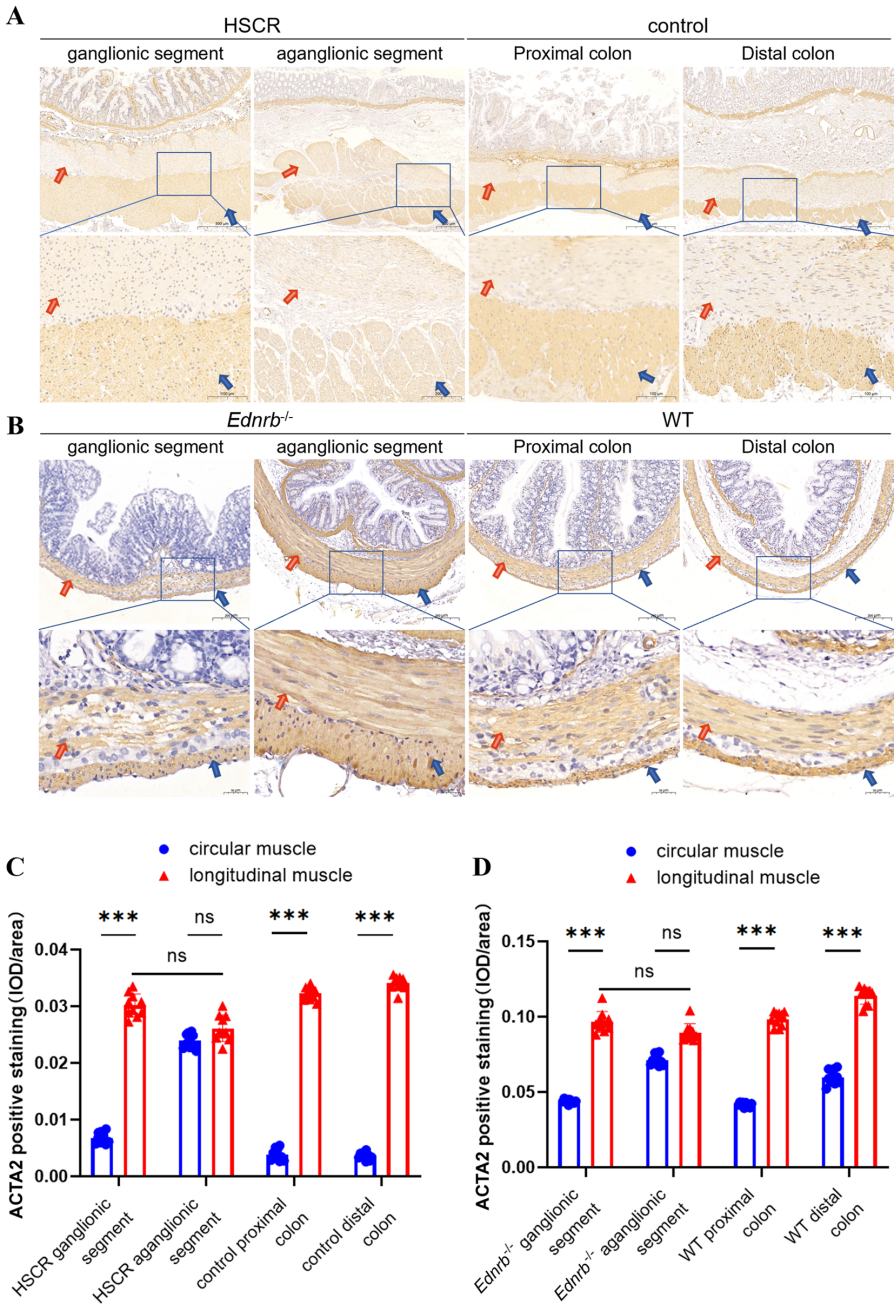
The expression of ACTA2 was different from control in HSCR patients and *Ednrb*^*−/−*^ mice. **A** Representative images of immunohistochemistry staining for ACTA2 in HSCR patient and age-matched normal control children colon tissues. **B** Representative images of immunohistochemistry staining for ACTA2 in P21d *Ednrb*^*−/−*^ mice and WT mice colon tissues. **C** The IOD/area value of ACTA2 positive expression of circular muscle and longitudinal muscle in HSCR patient and age-matched normal control children colon tissues. **D** The IOD/area value of ACTA2 positive expression of circular muscle and longitudinal muscle in P21d *Ednrb*^*−/−*^ mice and WT mice colon tissues. Values are the mean ± standard deviation. ns, not significance, ***p < 0.001 vs control group. IOD/area, Integrated optical density per stained area. The red arrows point to circular muscles and the blue ones point to longitudinal muscles

ACTA2 immunohistochemistry was used to stain colon tissue samples from HSCR patients and age-matched normal control children, the specific information of the HSCR patients is given in Table 1. In normal controls and the ganglionic segments of HSCR patients, the expression of ACTA2 was found to be much lower in circular SM than in longitudinal SM, and the difference was statistically significant. But in the aganglionic segments of HSCR patients, ACTA2 expression in circular SM was similar to that in longitudinal SM, with no statistical significance (Fig. 1A, C, Supplementary Fig. 2A). Consistent with the results of human patients, P21d WT mice and the ganglionic segments of *Ednrb*^*−/−*^ mice had a higher ACTA2 expression in longitudinal SM than in circular SM; the difference was statistically significant, and there was no difference in the aganglionic segments of *Ednrb*^*−/−*^ mice (Fig. 1B and D, Supplementary Fig. 2B). HE staining revealed ganglion cells between the circular and longitudinal muscles in the ganglionated segments of the intestinal canal but no ganglion cells in the aganglionated parts of the intestinal canal (Supplementary Fig. 1).

**Table 1:** HSCR patients’ information

HSCR cases number	Subtype	Gender	Age
1	Short-segment HSCR	Female	1 year 5 month
2	Short-segment HSCR	Male	3 year
3	Short-segment HSCR	Male	2 year 2 month
4	Short-segment HSCR	Male	4 year 6 month
5	Short-segment HSCR	Male	1 year 3 month
6	Long-segment HSCR	Female	8 month
7	Short-segment HSCR	Male	3 year 7 month
8	Short-segment HSCR	Male	1 year
9	Short-segment HSCR	Male	6 month

### Down regulation of *Acta2* weakens the contraction ability of intestinal smooth muscle cells

**Fig. 2:**
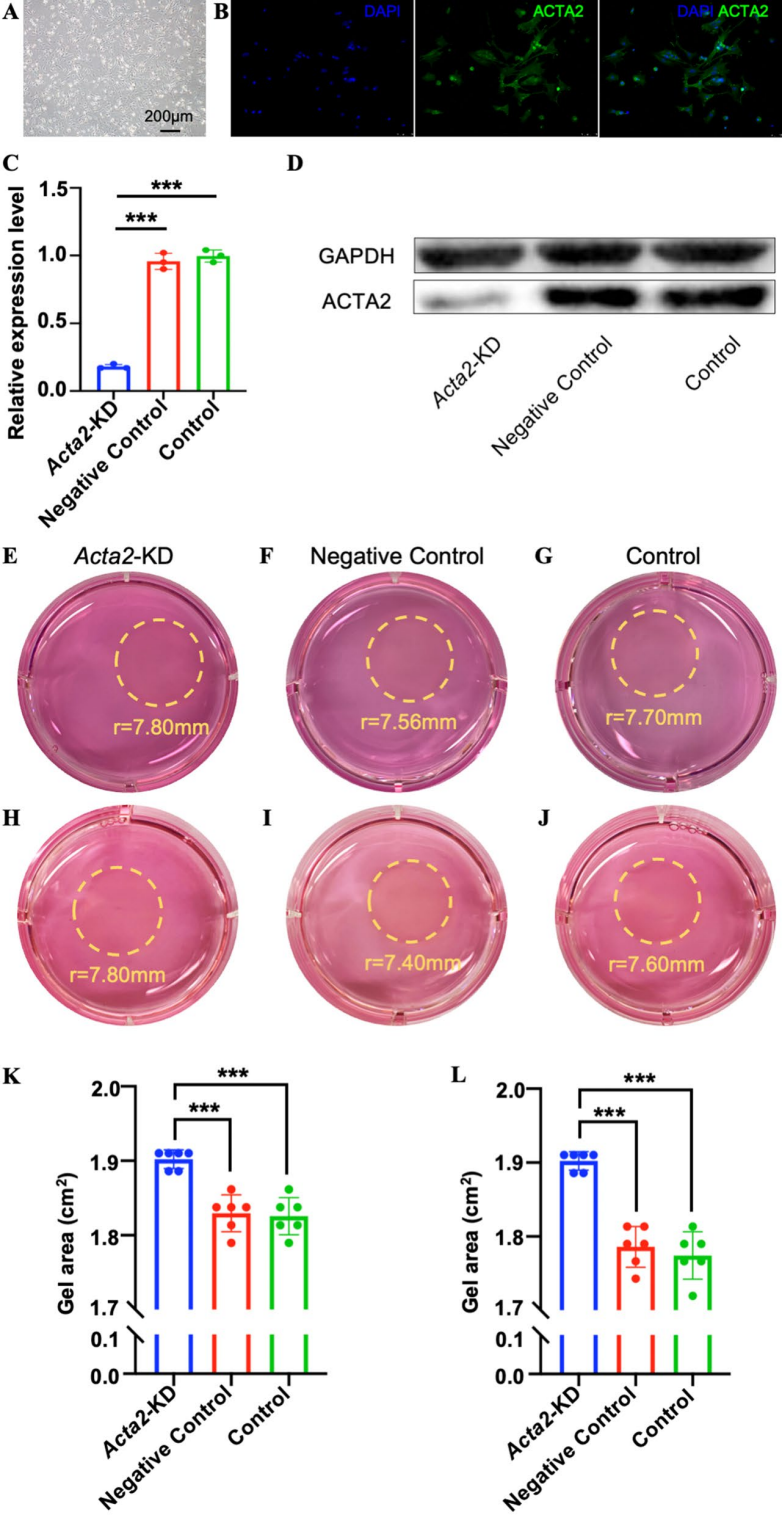
Down regulation of *Acta2* weakens the contraction ability of intestinal smooth muscle cells. **A** Morphology of primary intestinal smooth muscle cells. **B** Immunofluorescence microscopy of *Acta2* of primary intestine smooth muscle cells. **C** Relative expression level of *Acta2* mRNA in *Acta2* knockdown group, negative control group and blank control group. **D** Expression level of Acta2 protein in *Acta2* knockdown group, negative control group and blank control group. **E**–**J** Collagen gel contractility assays in smooth muscle cells of Acta2 knockdown group, negative control group and blank control group for 24 and 48 h. **K**, **L** Gel area of collagen matrices containing cells after 24 and 48 h, ***p < 0.001

Primary intestinal smooth muscle cells were extracted, cultured, and identified (Fig. 2A, B). siRNA technology was used to knock down *Acta2* expression; RT-qPCR and western blot were utilized to validate the efficiency of siRNA (Fig. 2C, D). The contractility of the *Acta2* knockdown group, the negative control group, and the blank control group was detected at 24 h and 48 h, and it was discovered that the *Acta2* knockdown group's contractility had significantly decreased at both 24 and 48 h (Fig. 2E, G, and K). The expression level of *Acta2* is related to smooth muscle function, and the contractility of smooth muscle is poor when the expression is low.

### The formation of circular muscles is later than longitudinal muscles, and the expression of ACTA2 in circular muscles is much lower than that in longitudinal muscles at different developmental stages

**Fig. 3:**
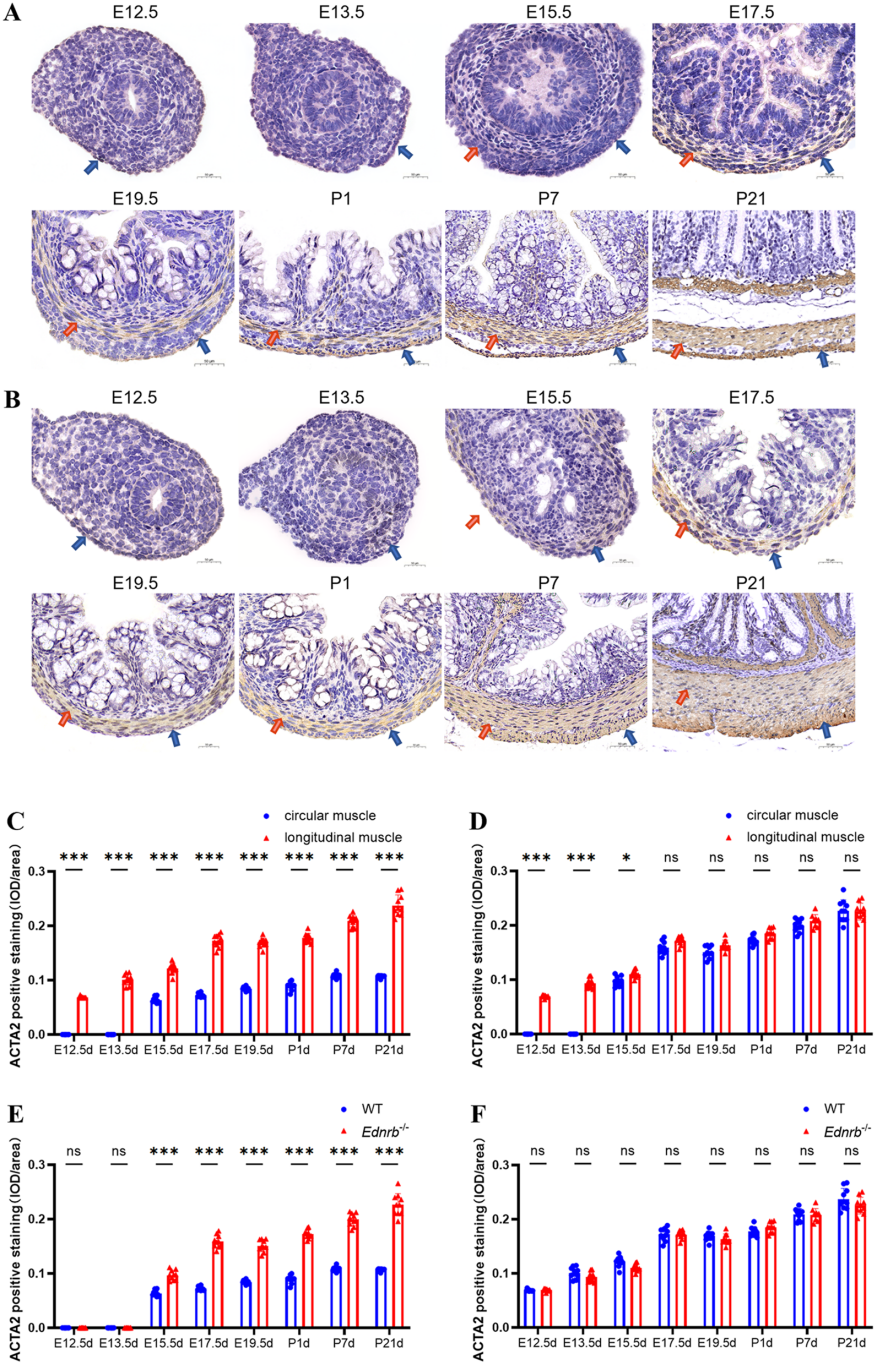
The relationship between the temporal and spatial expression of ACTA2 in the circular and longitudinal SM in *Ednrb*^*−/−*^ mice and WT mice. **A** Representative images of immunohistochemistry staining for ACTA2 in distal colon of WT mice at different life stage. **B** Representative images of immunohistochemistry staining for ACTA2 in distal colon of *Ednrb*^*−/−*^ mice at different development stage. **C** The IOD/area value of ACTA2 positive expression of circular SM and longitudinal SM in distal colon of WT mice at different development stage. **D** The IOD/area value of ACTA2 positive expression of circular SM and longitudinal SM in distal colon of *Ednrb*^*−/−*^ mice at different life stage. **E** The IOD/area value of ACTA2 positive expression of circular muscle in distal colon of *Ednrb*^*−/−*^ mice and WT mice at different development stage. **F** The IOD/area value of ACTA2 positive expression of longitudinal muscle in distal colon of *Ednrb*^*−/−*^ mice and WT mice at different development stage. Values are the mean ± standard deviation. *p < 0.05 vs control group; **p < 0.01 vs control group; ***p < 0.001 vs control group. IOD/area, Integrated optical density per stained area. The red arrows point to circular SM and the blue ones point to longitudinal SM

We further explored the relationship between the temporal and spatial expression of ACTA2 in the circular and longitudinal SM in WT mice. The distal colons of WT were stained for ACTA2 at E12.5d, E13.5d, E15.5d, E17.5d, E19.5d, P1d, P7d, and P21d (Fig. 3A and B). ACTA2-positive cells only occurred in the outer intestinal layers in WT mice at E12.5d and E13.5d and were found in both the inner (the region of future circluar muscle development) and outer (the region of future longitudinal muscle development) intestinal layers at E15.5d, E17.5d, E19.5d, P1d, P1w, and P3w, with ACTA2 expression being higher in the outer layer than the inner layer. The formation of circular SM is later than that of longitudinal SM in the distal colon.

### Abnormally elevated expression of ACTA2 of circular smooth muscle occurs since E15.5d in aganglionic segments of Ednrb^−/−^ mice

In *Ednrb*^*−/−*^ mice, ACTA2-positive cells were identified at the outer intestinal layers on E12.5d and E13.5d but not in the inner layer of the intestine. ACTA2-positive cells were found in the inner and outer layers of the intestine on E15.5d, E17.5d, E19.5d, P1d, P1w, and P3w. The formation of circular muscles is later than that of longitudinal muscles in the distal colon in *Ednrb*^*−/−*^ mice, which is similar to WT mice.

We separately compared the expression level of ACTA2 in the inner and outer layers of *Ednrb*^*−/−*^ and WT mice. According to the statistical results of the immunohistochemical images displayed, from E15.5d, the expression of ACTA2 in the inner layers of WT mice was lower than that in the outer layers, and the outcome was statistically significant (Fig. 3C, Supplementary Fig. 2C), whereas there was no statistically significant difference in ACTA2 expression between the inner and outer layers of *Ednrb*^*−/−*^ mice (Fig. 3D, Supplementary Fig. 2D), nor between the outer layers of *Ednrb*^*−/−*^ and WT mice (Fig. 3F), but ACTA2 expression in the inner layers of *Ednrb*^*−/−*^ mice was considerably higher than that in WT mice (Fig. 3E). In short, the expression of ACTA2 in the inner layers of *Ednrb*^*−/−*^ mice had been abnormally elevated since E15.5d.

As we all know, ENCCs moved to the proximal colon on E12.5d, the middle colon on E13.5d, and the final colon on E14.5d [31]. After the ENCCs are located in IMCs, persistent low expression of ACTA2 presents in the inner layers of the distal colon since E15.5d. However, we found higher expression of ACTA2 in the circular SM in the aganglionic segments of *Ednrb*^*−/−*^ mice. The absence of ENCCs may result in increased expression of ACTA2 in the circular SM.

## Discussion

The majority of children with HSCR are identified during infancy with symptoms of intestinal obstruction, such as failure to pass meconium in the first 24 h of life, progressive abdominal distention, difficult bowel movements, and so on [32]. Most disease-related symptoms arise because there is no propulsive motility (peristalsis, high-amplitude propagating contractions, or migrating motor complexes) in the aganglionic bowel and because the aganglionic bowel tonically contracts, making passage of stool and gas difficult [5, 33]. Our experimental results showed that the expression of ACTA2 is much higher in the longitudinal SM than the circular SM of WT mice and normal control children. Interestingly, we first found that the circular SM in aganglionic segments of *Ednrb*^*−/−*^ mice and HSCR patients exhibit increased expression of ACTA2. Therefore, the abnormal development of intestinal smooth muscle may be the causes of abnormal intestinal contractile function and thus intestinal spasm [6, 34].

Furthermore, we knocked down ACTA2 in mouse primary iSMCs to investigate the association between Acta2 and SM contractility. It was discovered that the expression level of Acta2 is related to smooth muscle function, and the contractility of smooth muscle is poor when the expression is low. Based on the findings, we speculated that the high expression of ACTA2 in the circular SM of HSCR aganglionic segments was to blame for the abnormally elevated contractility.

To investigate from which time point the aberrant expression of ACTA2 occurred, we compared the expression of ACTA2 in colon tissues from WT mice and *Ednrb*^*−/−*^ mice at various developmental stages. Experiment results turned out that under normal circumstances, after the ENCCs locate in colon (E15.5d), persistent low expression of ACTA2 presents in inner layers of distal colon, and the expression of ACTA2 in longitudinal SM is much higher than that of circular muscles cells, both in the embryonic and postnatal stages. Without ENCCs, however, the expression of ACTA2 in the inner layers in *Ednrb*^*−/−*^ mice was abnormally elevated since E15.5d and was similar to the outer layer. Some scholars have put up with the possible pathways that ENCCs regulate IMCs’ differentiation. Under the influence of ENCCs, most of IMCs first differentiated into C-KIT positive cells and then differentiated into SMC [24]. Above all, we believe that the abnormal expression of ACTA2 in HSCR is congenital and may be related to abnormal development of ENCCs, in other word, the lack of ENCCs may result in increased ACTA2 expression in circular SM.

The sensitivity, antigen concentration, and distribution density of immunohistochemical labeling will affect the positive staining intensity of samples. The higher the distribution density is and the brighter the color rendering of positive results is, the truer the results are. Immunohistochemistry, on the other hand, is semi-quantitative; hence, it might not be able to meet the demands of exact quantitative measurement. Although RT-qPCR and WB are effective techniques, it is difficult to separate circular muscle from longitudinal muscle. Single-cell spatial transcriptome quantitative analysis might be a more sophisticated method. In addition, there are still many mechanisms to be studied, such as how the expression level of ACTA2 influences the contractility of smooth muscle and the signaling pathway by which smooth muscle spasm occurs.

### Supplementary Information

Below is the link to the electronic supplementary material.

Supplementary file1 (TIFF 6109 KB)

Supplementary file2 (TIFF 6875 KB)

## Data Availability

The authors confirm that the data supporting the findings of this study are available within the article.

## References

[1] Kyrklund K, Sloots CEJ, de Blaauw I, Bjornland K, Rolle U, Cavalieri D (2020). ERNICA guidelines for the management of rectosigmoid Hirschsprung’s disease. Orphanet J Rare Dis.

[2] Soret R, Schneider S, Bernas G, Christophers B, Souchkova O, Charrier B (2020). Glial cell-derived neurotrophic factor induces enteric neurogenesis and improves colon structure and function in mouse models of Hirschsprung disease. Gastroenterology.

[3] Butler Tjaden NE, Trainor PA (2013). The developmental etiology and pathogenesis of Hirschsprung disease. Transl Res.

[4] Chen X, Meng X, Zhang H, Feng C, Wang B, Li N (2020). Intestinal proinflammatory macrophages induce a phenotypic switch in interstitial cells of Cajal. J Clin Invest.

[5] Heuckeroth RO (2018). Hirschsprung disease - integrating basic science and clinical medicine to improve outcomes. Nat Rev Gastroenterol Hepatol.

[6] Matsuda H, Hirato J, Kuroiwa M, Nakazato Y (2006). Histopathological and immunohistochemical study of the enteric innervations among various types of aganglionoses including isolated and syndromic Hirschsprung disease. Neuropathology.

[7] Vizi ES, Zséli J, Kontor E, Feher E, Verebélyi T (1990). Characteristics of cholinergic neuroeffector transmission of ganglionic and aganglionic colon in Hirschsprung's disease. Gut.

[8] Le TL, Galmiche L, Levy J, Suwannarat P, Hellebrekers DM, Morarach K (2021). Dysregulation of the NRG1/ERBB pathway causes a developmental disorder with gastrointestinal dysmotility in humans. J Clin Invest.

[9] Arnoldi R, Hiltbrunner A, Dugina V, Tille JC, Chaponnier C (2013). Smooth muscle actin isoforms: a tug of war between contraction and compliance. Eur J Cell Biol.

[10] Kedinger M, Simon-Assmann P, Bouziges F, Arnold C, Alexandre E, Haffen K (1990). Smooth muscle actin expression during rat gut development and induction in fetal skin fibroblastic cells associated with intestinal embryonic epithelium. Differentiation.

[11] Chen H, Li X, Gao L, Zhang D, Han T (2022). Construction and identification of an immortalized rat intestinal smooth muscle cell line. Neurogastroenterol Motil.

[12] Huycke TR, Miller BM, Gill HK, Nerurkar NL, Sprinzak D, Mahadevan L (2019). Genetic and mechanical regulation of intestinal smooth muscle development. Cell.

[13] McHugh KM (1995). Molecular analysis of smooth muscle development in the mouse. Dev Dyn.

[14] Li C, Vu K, Hazelgrove K, Kuemmerle JF (2015). Increased IGF-IEc expression and mechano-growth factor production in intestinal muscle of fibrostenotic Crohn's disease and smooth muscle hypertrophy. Am J Physiol Gastrointest Liver Physiol.

[15] Liu X, Lui VCH, Wang H, Ye M, Fan R, Xie X (2022). Temporal and spatial development of intestinal smooth muscle layers of human embryos and fetuses. J Dev Orig Health Dis.

[16] Hao MM, Foong JP, Bornstein JC, Li ZL, Vanden Berghe P, Boesmans W (2016). Enteric nervous system assembly: Functional integration within the developing gut. Dev Biol.

[17] Torihashi S, Ward SM, Sanders KM (1997). Development of c-Kit-positive cells and the onset of electrical rhythmicity in murine small intestine. Gastroenterology.

[18] Fawkner-Corbett D, Antanaviciute A, Parikh K, Jagielowicz M, Gerós AS, Gupta T (2021). Spatiotemporal analysis of human intestinal development at single-cell resolution. Cell.

[19] Qiu H, Zhu Y, Sun Z, Trzeciakowski JP, Gansner M, Depre C (2010). Short communication: vascular smooth muscle cell stiffness as a mechanism for increased aortic stiffness with aging. Circ Res.

[20] Ibrahim MM, Chen L, Bond JE, Medina MA, Ren L, Kokosis G (2015). Myofibroblasts contribute to but are not necessary for wound contraction. Lab Invest.

[21] Wang J, Meng X, Feng C, Xiao J, Zhao X, Xiong B (2021). Benzophenone-3 induced abnormal development of enteric nervous system in zebrafish through MAPK/ERK signaling pathway. Chemosphere.

[22] Xiao J, Hao LW, Wang J, Yu XS, You JY, Li ZJ (2023). Comprehensive characterization of the genetic landscape of familial Hirschsprung's disease. World J Pediatr.

[23] Tang CS, Karim A, Zhong Y, Chung PH, Tam PK (2023). Genetics of Hirschsprung's disease. Pediatr Surg Int.

[24] Radenkovic G, Radenkovic D, Velickov A (2018). Development of interstitial cells of Cajal in the human digestive tract as the result of reciprocal induction of mesenchymal and neural crest cells. J Cell Mol Med.

[25] Jia XL, Li SY, Dang SS, Cheng YA, Zhang X, Wang WJ (2012). Increased expression of chondroitin sulphate proteoglycans in rat hepatocellular carcinoma tissues. World J Gastroenterol.

[26] Aasebø K, Dragomir A, Sundström M, Mezheyeuski A, Edqvist PH, Eide GE (2020). CDX2: a prognostic marker in metastatic colorectal cancer defining a better BRAF mutated and a worse KRAS mutated subgroup. Front Oncol.

[27] Wang H, Li Y, Zhou D, Li X, Jia S, Qi S (2021). Aldehyde dehydrogenase 1B1 is a potential marker of colorectal tumors. Histol Histopathol.

[28] Murthy KS, Makhlouf GM (1995). Interaction of cA-kinase and cG-kinase in mediating relaxation of dispersed smooth muscle cells. Am J Physiol.

[29] Murthy KS, Jin JG, Grider JR, Makhlouf GM (1997). Characterization of PACAP receptors and signaling pathways in rabbit gastric muscle cells. Am J Physiol.

[30] Teng B, Murthy KS, Kuemmerle JF, Grider JR, Sase K, Michel T (1998). Expression of endothelial nitric oxide synthase in human and rabbit gastrointestinal smooth muscle cells. Am J Physiol.

[31] Nagy N, Goldstein AM (2017). Enteric nervous system development: a crest cell's journey from neural tube to colon. Semin Cell Dev Biol.

[32] Kessmann J (2006). Hirschsprung's disease: diagnosis and management. Am Fam Physician.

[33] Swenson O, Rheinlander HF, Diamond I (1949). Hirschsprung's disease; a new concept of the etiology; operative results in 34 patients. N Engl J Med.

[34] Xiao J, Meng X, Chen K, Wang J, Wu L, Chen Y (2022). Down-regulation of double C2 domain alpha promotes the formation of hyperplastic nerve fibers in aganglionic segments of Hirschsprung's disease. Int J Mol Sci.

