# Supplementary figures and images for: Abnormally elevated expression of ACTA2 of circular smooth muscle leads to hyperactive contraction in aganglionic segments of HSCR
Source: Pediatr Surg Int. 2023 Jun 6;39(1):214. doi: 10.1007/s00383-023-05479-x (PMC10244273; doi:10.1007/s00383-023-05479-x)

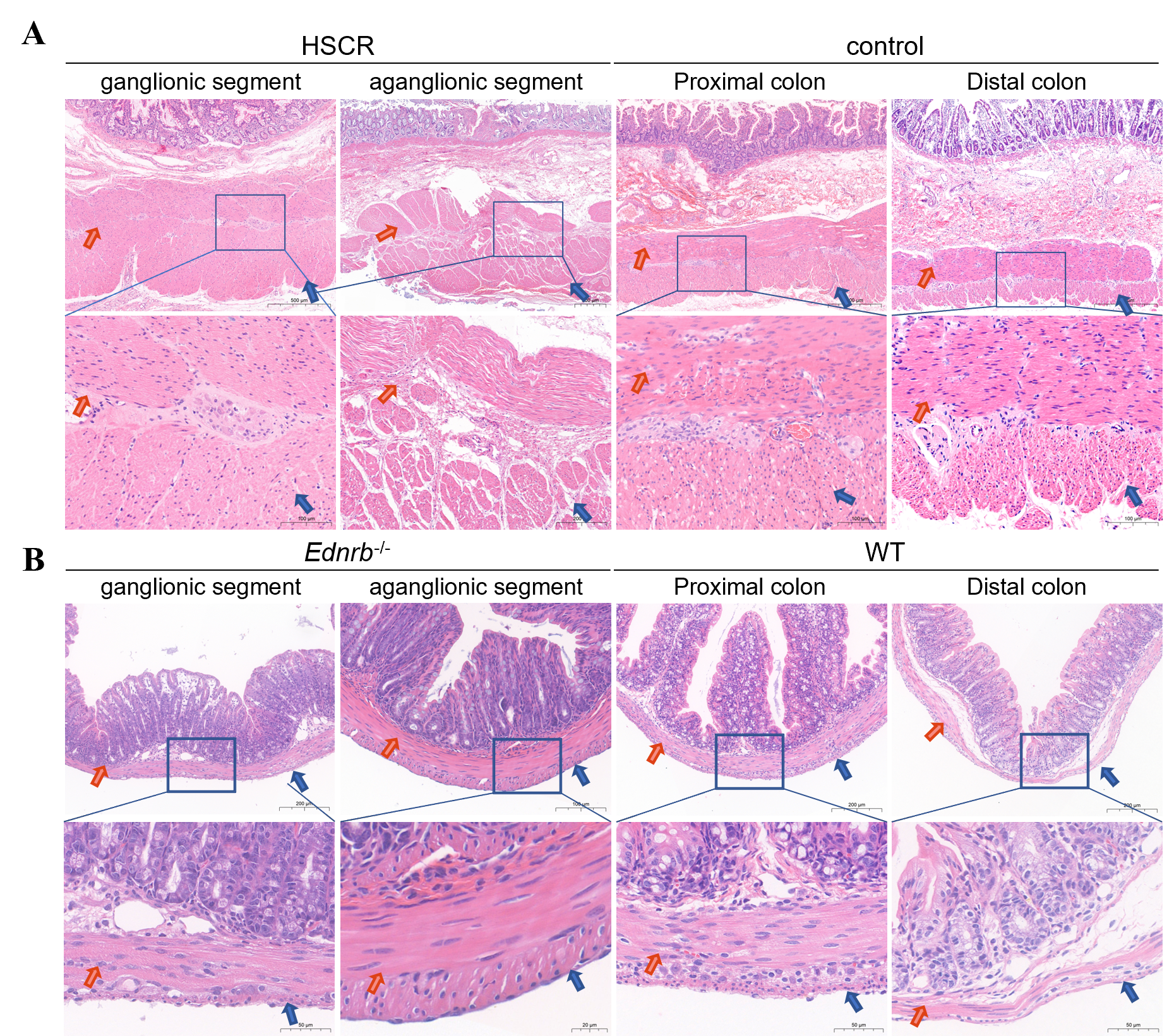

Supplement: Supplementary file 1 — Supplementary file1 (TIFF 6109 KB) [file 383_2023_5479_MOESM1_ESM.tiff]

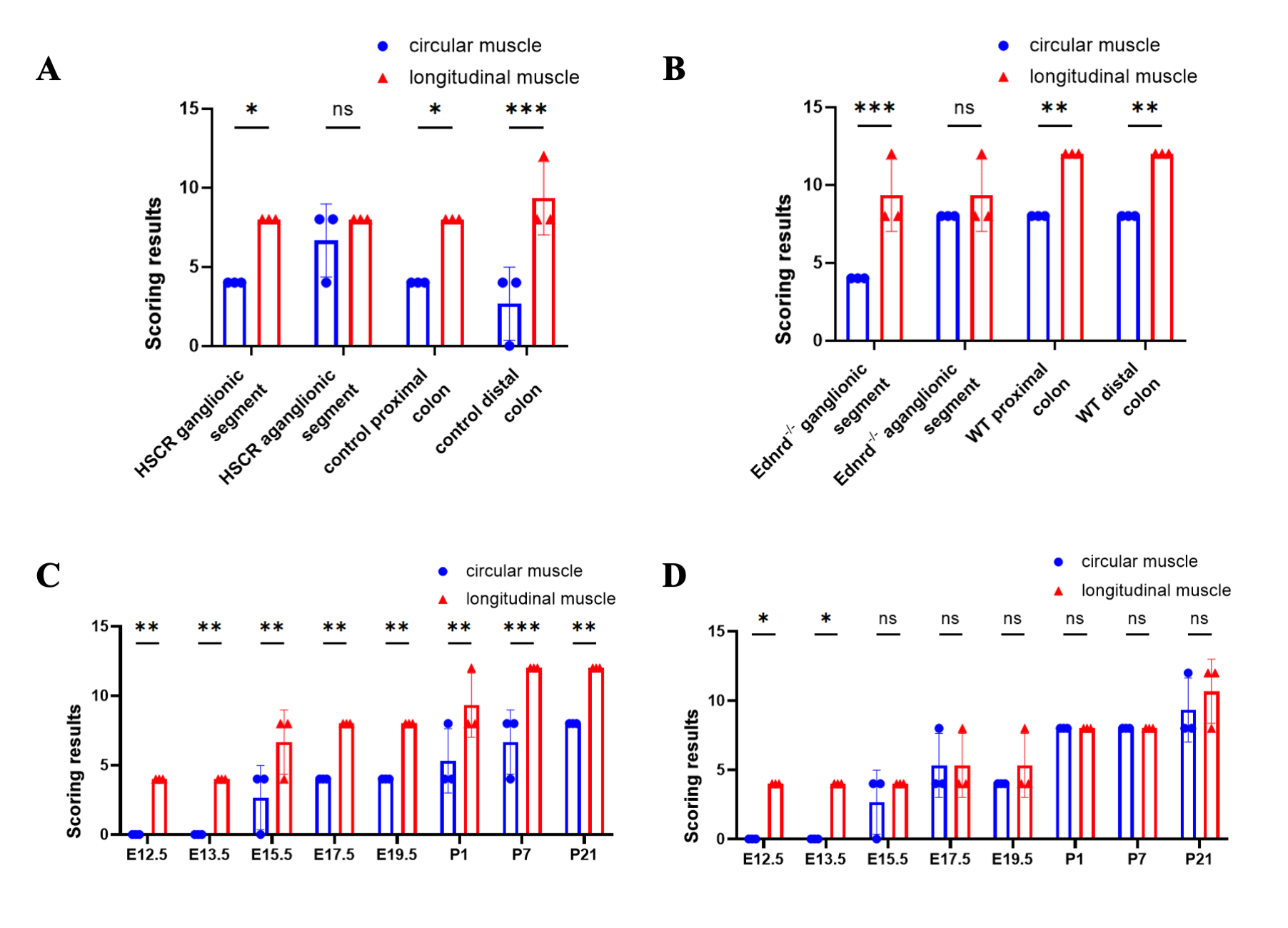

Supplement: Supplementary file 2 — Supplementary file2 (TIFF 6875 KB) [file 383_2023_5479_MOESM2_ESM.tiff]
